# Symptoms of anxiety and depression in adolescent students; a perspective from Sri Lanka

**DOI:** 10.1186/1753-2000-4-10

**Published:** 2010-03-24

**Authors:** Chaturaka Rodrigo, Srina Welgama, Jayantha Gurusinghe, Thilina Wijeratne, Gamini Jayananda, Senaka Rajapakse

**Affiliations:** 1 Psychiatry Unit, Provincial General Hospital, Ratnapura, Sri Lanka; 2 Department of Clinical Medicine, Faculty of Medicine, University of Colombo, Sri Lanka

## Abstract

**Background:**

Sri Lanka recorded an extraordinary high suicide rate for adolescents aged 15 - 19 in the early 1990s (46.5/100,000). With this in perspective, the Ministry of Health in Sri Lanka recommends school programmes for adolescents by mental health units of local hospitals.

**Methods:**

We conducted cross sectional surveys to screen for symptoms of anxiety and depression among students aged 14 - 18 during school mental health programmes. Two schools were randomly selected within the Ratnapura municipality (urban population of approx. 50,000), Sri Lanka and all students aged 14-18 were assessed with self administered (pre tested, Sinhalese translations) questionnaires [Center for epidemiologic studies depression scale, Anxiety screening test of suicide and mental health association international].

**Results:**

A total of 445 students were assessed (male-54.4%, female 45.6%). Thirty six percent screened positive for depression (mild depression-17%, severe depression-19%) and 28% screened positive for severe anxiety. Females screened positive for depression and anxiety significantly more than the males (p = 0.0001, 0.005 respectively). Students in classes facing barrier examinations at the end of the year had the highest positivity rates. Examination related issues (36%) were the most commonly cited problem.

**Recommendations:**

It is recommended that:

1. School mental health development programmes in Sri Lanka concentrate more on reducing examination related stress, and in particular focus on the female students

2. Policy decisions are made to reduce competition for higher education

3. A nationally coordinated survey on mental health of adolescent students is carried out utilizing the island-wide network of medical officers of mental health.

## Letter to editor

Sir: Published research on adolescent psychiatry in Sri Lanka is minimal. Unfortunately, Sri Lanka also recorded an extraordinary high suicide rate for adolescents aged 15 - 19 in the early 1990s(46.5/100,000)[1,2]. With this in perspective, the Ministry of Health in Sri Lanka recommends school programmes for adolescents by mental health units of local hospitals.

The objectives of this study were to

1. screen for symptoms of anxiety and depression in a sample of adolescent students

2. identify the issues affecting the mental health of adolescents

3. demonstrate the relevance of a countrywide mental health assessment of adolescents in concurrence with school mental health programmes

We conducted cross sectional surveys to screen for symptoms of anxiety and depression among students aged 14 - 18 during school mental health programmes. Two schools were randomly selected within the Ratnapura municipality (urban population of approx. 50,000), Sri Lanka and all students aged 14-18 were assessed with self administered (pre tested, Sinhalese translations) questionnaires [Center for epidemiologic studies depression scale (CES-D), Anxiety screening test of suicide and mental health association international][3,4]. Statistical significances were calculated with chi square test. Permission for the study was granted by the Provincial Department of Education. Verbal consent was obtained from participants in classroom and only the consenting students filled the questionnaire. Contribution from participants was anonymous.

A total of 445 students were assessed (male-54.4%, female 45.6%). Thirty six percent screened positive for depression (mild depression-17%, severe depression-19%) and 28% screened positive for severe anxiety (Table 1). Females screened positive for depression and anxiety significantly more than the males (p = 0.0001, 0.005 respectively). While there were no differences between grade 9 (aged 14) and 10 (aged 15) students, grade 11(aged 16) students had significantly high rates of depression and severe anxiety (p < 0.00001, 0.0001 respectively). Numbers in grades 12 (aged 17) and 13 (aged 18) were small for a valid analysis but proportion-wise, grade 13 had the second highest depression and anxiety scores. Examination related issues (36%) were the most commonly cited problem (Table 2).

**Table 1:** Percentages (%) of students in each grade with symptoms and anxiety and depression*

Grade	Percentages (%)					
	**No depression**	**Mild depression**	**Severe depression**	**No anxiety**	**Mild Anxiety**	**Severe Anxiety**

Grade 9 (n - 101)	63.4	15.8	18.8	13.9	62.4	21.8

Grade 10 (n - 150)	74.0	14.0	10.0	12.7	64.7	21.3

Grade 11 (n - 131)	47.3	26.0	25.2	8.4	51.1	40.5

Grade 12 (n - 35)	65.7	11.4	22.9	2.8	68.6	28.6

Grade 13 (n - 28)	64.3	7.1	28.6	14.3	53.6	32.1

*Seven (1.6%) and four (0.9%) students did not complete the depression and anxiety questionnaires respectively. Grades 9,10,11,12,13 corresponds to ages 14,15,16,17 and 18 respectively.

The cut off values for CESD scale and the anxiety screening test are as follows;

Score of CES - D scale: less than 15; no depression, 16-21; mild depression, more than 21; possibility of severe depression[5]

Score of anxiety screening test: 0; no anxiety, 1-4; mild anxiety, 5-10; severe anxiety[4]

**Table 2:** Prominent issues cited by respondents to affect their mental health and the relevant percentages in relation to the total sample

Problem	Number*	Percentage
Difficulty in studying	133	29.9

Fear of Examination	27	6.1

Anger management	20	4.5

Problems with teachers	14	3.1

Frequent scolding by parents	10	2.2

Problems with romantic partners	8	1.8

Loneliness	7	1.6

Problems with friends	6	1.3

Physical symptoms	6	1.3

*Not all students had answered this section and some had indicated more than one problem

This survey shows that:

1. a significant proportion of adolescents suffer from symptoms of anxiety and depression

2. these symptoms are mainly attributable to examination induced stress

The Government of Sri Lanka provides free education in all public schools. However, given the limited resources, access to better schools, and universities is subject to severe competition. There are two important barrier examinations for a student in Sri Lanka; the General Certificate of Education (G.C.E) - Ordinary level examination which determines entrance to advanced level classes (held at the end of grade 11) and the G.C.E-Advanced level examination which determines university entrance (held at the end of grade 13). The symptoms of anxiety and depression were more among students in these classes.

Surprisingly, issues with romantic partners and drug addiction did not surface prominently. However, it is possible that students did not reveal such issues, since the subjects are often culturally taboo. Our personal experience is that many adolescents with deliberate self harm (DSH) referred to us have done so due to problems with family or romantic partners. Since many appear to have background symptoms of depression, additional life stressors may easily push vulnerable adolescents towards DSH.

We conclude that:

1. A significant proportion of adolescents aged 14 - 18 suffer from symptoms of anxiety and depression. The main identified cause was examination related stress

2. Females are significantly more symptomatic than males

It is recommended that:

1. School mental health development programmes in Sri Lanka concentrate more on reducing examination related stress, and in particular focus on the female students

2. Policy decisions are made to reduce competition for higher education

3. A nationally coordinated survey on mental health of adolescent students is carried out utilizing the island-wide network of medical officers of mental health.

## Competing interests

The authors declare that they have no competing interests.

## Authors' contributions

All authors have participated in designing, executing, data analysis and writing of the manuscript. All authors have read and approved the final manuscript

## Authors' information

CR, SW and JG are medical officers of mental health attached to the psychiatry unit of Provincial General Hospital, Ratnapura. TW is the psychiatric social worker of the unit and GJ is the consultant psychiatrist of the unit. SR is the head and senior lecturer of the Department of Clinical Medicine, Faculty of Medicine, University of Colombo, Sri Lanka
